# Blended host ink for solution processing high performance phosphorescent OLEDs

**DOI:** 10.1038/s41598-019-43359-4

**Published:** 2019-05-02

**Authors:** Tong Lin, Xue Sun, Yongxu Hu, Wanying Mu, Yuling Sun, Dongyu Zhang, Zisheng Su, Bei Chu, Zheng Cui

**Affiliations:** 1 Printable electronics research center, Suzhou Institute of nanotech and nano-bionics, Chinese Academy of Sciences, Suzhou, 215123 Jiangsu P.R. China; 2 State Key Laboratory of Luminescence and Applications, Changchun Institute of Optics, Fine Mechanics and Physics, Chinese Academy of Sciences, Changchun, 130033 P.R. China; 3 School of Chemical Engineering, University of Science and Technology Liaoning (USTL), Anshan, 114051 P.R. China; 4 College of Materials Science and Engineering, Shanghai University, Shanghai, 200444 P.R. China; 5 College of Physics and Information Engineering, Quanzhou Normal University, Quanzhou, 362000 P.R. China

**Keywords:** Organic LEDs, Photonic devices

## Abstract

In order to solve the interface issues in solution deposition of multilayer OLED devices, a blended host concept was developed and applied to both spin-coating and inkjet printing of phosphorescent OLEDs. The blended host consists of 1,3-bis(carbazolyl)benzene (mCP) and1,3,5-tri(phenyl-2-benzimidazoly)-benzene (TPBi). Maximum current efficiency (CE) of 24.2 cd A^−1^ and external quantum efficiency (EQE) of 7.0% have been achieved for spin-coated device. Maximum CE and EQE of 23.0 cd A^−1^ and 6.7% have been achieved for inkjet printed device. The films deposited by printing and spin-casting were further researched to explore the effect of those different processing methods on device performance.

## Introduction

Organic light-emitting diodes (OLEDs) have been drawing record attentions in the past few years, because of the industrialization of OLED mobile phones and TVs and potentials in many other applications^1–6^. Although thermal evaporation is the dominant process to deposit OLED materials, the high cost associated with the process, particularly the large waste of expensive OLED materials through shadow masks, has been the major factor to deter the widespread use of this technology. In responding to this problem, deposition of OLED materials by solution process is being developed in recent years. The solution process, either spin-coating which is suitable for making lighting panels or inkjet printing which is suitable for making display panels, can significantly reduce material waste and the process complexity, which can lead to significant reduction of manufacturing cost. However, solution process has its own issues compared to traditional thermal evaporation process. For example, OLED materials have to be converted into printable inks and the ink surface tension, viscosity, solvent density, solubility and solvent evaporation rate have to be in good match to the requirements of printing methods. The recrystallization, phase separation, and coffee-ring effect, associated with ink drying process have serious impact on the formation of uniform films. The interface between printed function layers severely influences the performance of device. Hence, the solution processed OLEDs are not as good as thermally evaporated devices. Within the solution process, inkjet-printed devices are usually worse than the spin-coated devices^7–9^. As a result, the most of reported OLEDs by inkjet printing are a single printing layer structure, either hole transporting layer (HTL)^10–16^ or emission layer (EML)^17–20^, because bilayer printed OLEDs always show inferior performance^17–19^. Conventional thermal evaporation process has been using blended host materials (co-evaporation of multi-materials) for OLEDs to achieve balanced carrier transport^20,21^. This concept could be applied to solution process to solve the interface issue happened in solution deposition of multilayer OLED devices. Compared to thermal evaporation process, the ratio of individual component in the blended host could be controlled precisely and easily in solution process.

In this paper, a blended host solution is reported for spin-coating of emissive layer (EML) in OLED, which consists of 1,3-bis(carbazolyl)benzene (mCP) blended with1,3,5-tri(phenyl-2-benzimidazoly)-benzene (TPBi) as the host and tris[2-(p-tolyl)pyridine]iridium(III) (Ir(mppy)_3_) as the dopant with 45:45:10 ratio. Maximum current efficiency (CE) of 24.2 cd A^−1^ and external quantum efficiency (EQE) of 7.0% have been achieved for spin-coated device. The same blended host ink was used in inkjet printing of OLED and the printed device showed the performance comparable to the best spin-coated devices.

## Results and Discussion

**Figure 1:**
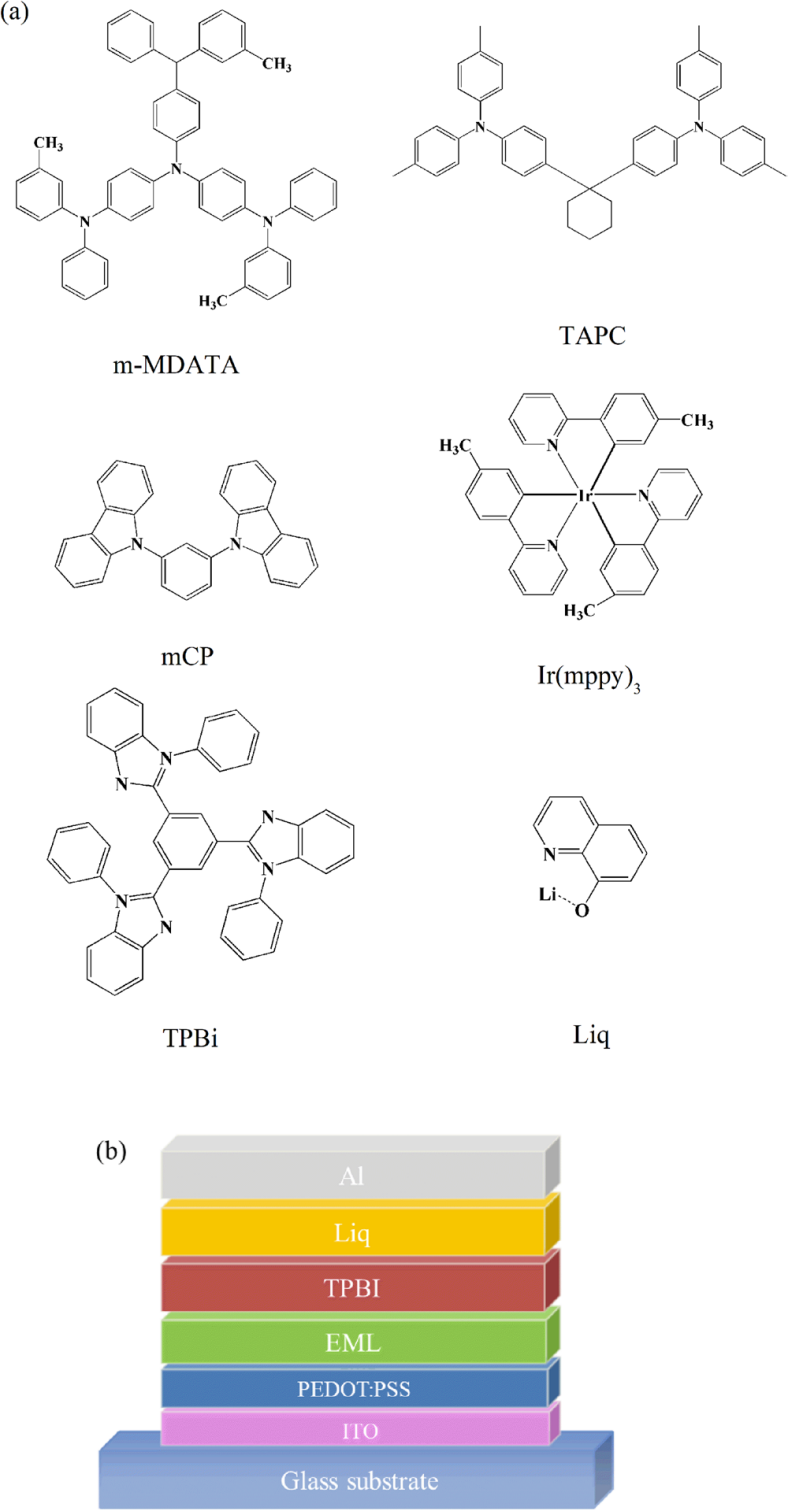
Molecular structure and device configuration.

The chemical structure of all organic materials used in this work is depicted in Fig. 1a. Due to their wide energy gaps and appropriate solubility in many solvents, mCP, 4,4′,4″-tris[3-methylphenyl(phenyl)- aminotriphenylamine (m-MTDATA), 1,1-bis((di-4-tolylamino)phenyl)cyclohexane (TAPC) and TPBi were selected as candidates of donor and acceptor for the blend host, respectively. Ir(mppy)_3_ and 8-hydroxyquinolatolithium (Liq) were used as emission and electron injection materials. The OLEDs structure is shown in Fig. 1b, which consisted of ITO/PEDOT:PSS (25 nm)/emissive layer (25 nm)/TPBi (30 nm)/Liq (1 nm)/Al (100 nm). The thickness of PEDOT:PSS and emissive layer (EML) was kept at about 25 nm for both spin-coating and inkjet printing, in order to compare the performance of devices made by different solution processing methods.

**Figure 2:**
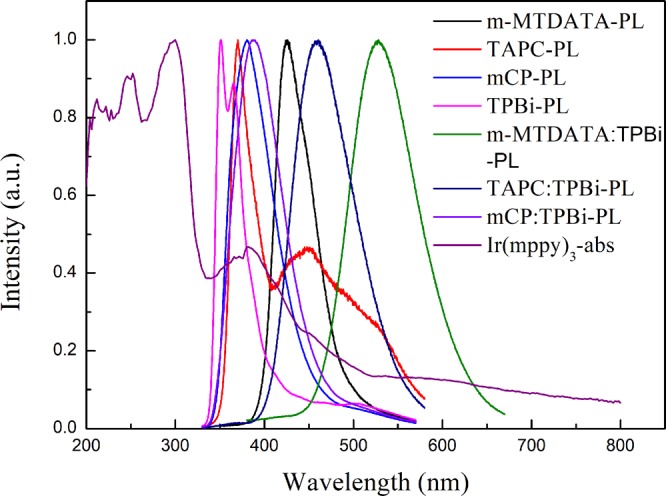
PL spectra of m-MTDATA, TAPC, mCP, TPBi and their mixtures and the absorption of Ir(mppy)_3_.

Figure 2 shows the PL spectra of m-MTDATA, TAPC, mCP, TPBi and their mixtures, as well as the absorption spectrum of Ir(mppy)_3_ films. The peak at 380 nm is for mCP, while 351 nm for TPBi. However, the broad PL of mCP: TPBi film with a peak at 388 nm, which is red-shifted relative to those components, can be attributed to the exciplex formation between mCP: TPBi^22^. It is the same as m-MTDATA:TPBi and TAPC:TPBi to form exciplexes^23,24^. Moreover, there is significant overlap between the absorption of Ir(mppy)_3_ and PL spectra of mCP: TPBi, implying that the energy of exciplex can efficiently transfer to the phosphor dopant. However, it is hard for m-MTDATA:TPBi and TAPC:TPBi to transfer energy to the phosphor dopant because of the small overlap between the absorption of Ir(mppy)_3_ and PL spectra of relative exciplex. The OLED devices were made with the three blended host materials (mCP: TPBi, m-MTDATA:TPBi and TAPC:TPBi) and their performances were listed in Table S1 and shown in Fig. S1. The device with mCP:TPBi blended host got higher efficiency than the m-MTDATA:TPBi and TAPC:TPBi based devices. Therefore, mCP: TPBi was chosen as the blended host in this work.

To achieve printable inkjet, many parameters, such as viscosity, surface tension, and density have to be considered^24,25^. The characteristic number Z is always used to predict the stable droplet formation, which is determined as follows:

$${\rm{z}}=\frac{\sqrt{d\rho \gamma }}{\eta }$$

where d is the diameter of jetting nozzle for inkjet printing. *ρ*, *γ*, and *η* are the density, surface tension, and viscosity of inks, respectively^26^. In general, the Z for stable inkjet-printing is expected between 1 and 10^25–27^. The properties of various solvents used in this work are shown in Table 1. HTL-Ink is used for HTL printing, which consists of PEDOT:PSS and ethylene glycol with the ratio of 1:3. The solvents of EML-1~5 include 5% CB and 95% butyl benzoate with different ratios of hosts. The Z of HTL-Ink and EML-1~5 varies from 1.8 to 12.5, which is within or close to the requirement range of printable ink. Practically, experiments demonstrate that all of the inks can be printed smoothly. The boiling point is another vital parameter for ink-jetting process. The primary solvent for PEDOT:PSS is water, whose boiling point is at 100 °C. When PEDOT:PSS is being printed, the previous printed parts start to dry before completion of printing procedure, which caused poor uniformity of films. To solve this problem, ethylene glycol with boiling point of 197 °C is added into the HTL-Ink. As for EML inks, the butyl benzoate is chosen to be the primary solvent because of its suitable Z and high boiling point of 250 °C. The solubility of it for mCP, TPBi and Ir(mppy)3 are 30, 17 and 0.5 mg/ml, respectively. To further enhance the solubility, especially for Ir(mppy)3, chlorobenzene (CB) is chosen to be secondary solvent. The details of EML-1~5 solutes have been shown in Table 2. Interestingly, there is no remarkable difference of properties among EML-1 to 5, which means there is little effect on Z with different solute ratios.

**Table 1:** Properties of the solvents^a^.

Solvent	Boiling point (°C)	Viscosity (cp)	Surface tension (mN m^−1^)	Density (g cm^−3^)	Z
PEDOT:PSS	100	7.40	65.7	1.03	5.1
Ethylene glycol	197	14.83	47.9	1.14	2.3
HTL-Ink^b^	—	20.00	52.3	1.13	1.8
CB	132	0.76	33.6	1.11	36.7
Butyl Benzoate	250	2.70	33.4	1.01	8.7
EML-1~5^c^	—	2.11	33.6	1.03	12.5

^a^Data measured at 25 °C.

^b^HTL-Ink consists of 25% PEDOT:PSS and 75% ethylene glycol.

^c^EML-1~5 solvents consist of 5% CB and 95% butyl benzoate.

**Table 2:** The design of OLED devices.

	PEDOT:PSS	EML(mCP:TPBi:Ir(mppy)_3_)
Device A	Spin-coating	Printing EML-1 (30:60:10)
Device B	Spin-coating	Printing EML-2 (45:45:10)
Device C	Spin-coating	Printing EML-3 (60:30:10)
Device D	Spin-coating	Printing EML-4 (75:15:10)
Device E	Spin-coating	Printing EML-5 (90:0:10)
Device F	Spin-coating	Spin-coating (45:45:10)
Device G	Printing	Spin-coating (45:45:10)
Device H	Printing	Printing EML-2 (45:45:10)
Device I	Spin-coating	Spin-coating (90:0:10)

**Figure 3:**
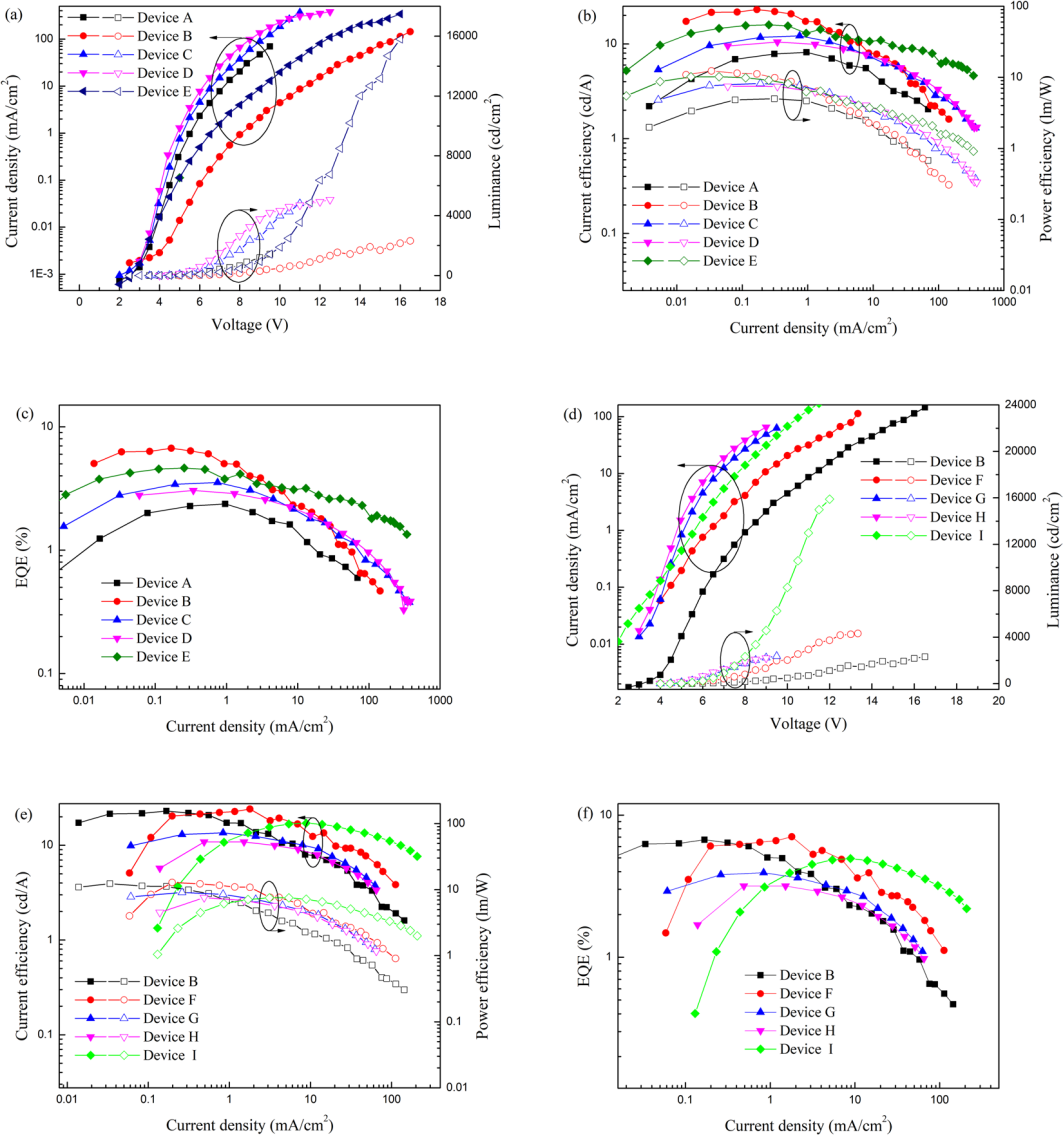
(**a**,**d**) V-J-L curves, (**b**,**e**) J-EQE curves, and (**c**,**f**) J-CE-PE curves of Device A-I.

A series of OLEDs (Device A-I) were designed and constructed for comparison in this work, as shown in Table 2. The HIL and EML were fabricated by spin-coating or printing, the electron transporting layer (ETL) electron injection layer (EIL) and cathode were deposited by thermal evaporation. The Voltage (V)-current density (J)-luminance (L), J-external quantum efficiency (EQE), and J-current efficiency (CE)-power efficiency (PE) curves of Device A-I are shown in Fig. 3 and the data are summarized in Table 3. The data indicate that the turn-on voltage is around 4 V for all the devices, which is lower than most of the printed OLEDs reported in literatures^7,8,14,16^.

**Table 3:** The performance of OLED devices.

	V_trun-on_^a^ (V)	L_max_^b^ (cd m^−2^)	CE/PE/EQE (cd A^−1^/lm W^−1^/%)		
			Maximum	@100 cd cm^−2^	@1000 cd cm^−2^
Device A	4.1	1422	8.2/5.0/2.3	7.5/4.0/2.2	2.8/0.9/0.8
Device B	4.0	2314	23.0/12.3/6.7	20.9/9.1/6.1	7.4/2.0/2.2
Device C	3.8	4812	12.2/8.2/3.5	11.9/7.4/3.5	6.2/2.8/1.8
Device D	3.7	5067	10.5/7.5/3.0	10.1/6.4/2.9	6.6/3.2/1.9
Device E	3.7	15800	15.9/10.2/4.6	14.3/7.2/4.2	10.7/3.7/3.1
Device F	3.7	4324	24.2/12.8/7.0	21.4/12.2/6.2	16.8/6.2/4.9
Device G	3.6	2366	13.5/9.1/3.9	13.5/8.5/3.9	9.2/4.1/2.7
Device H	3.5	2192	10.9/7.6/3.2	10.8/7.2/3.2	8.0/3.8/2.3
Device I	3.8	15880	17.1/7.5/4.9	10.8/6.2/3.1	16.8/7.5/4.8

^a^The voltage at 1 cd m^−2^. ^b^Maximum luminance.

For exciplex OLEDs, the best ratio of donor and acceptor is not always 1:1 in our previous work^23,28,29^. To attain moderate proportion of donor and acceptor, the Device A-E are designed with the same spin-coating PEDOT:PSS and varied content ratios between mCP and TPBi. The best inkjet-printed Device B (mCP, TPBi and Ir(mppy)_3_ at ratio of 45:45:10) achieved maximum CE, PE and EQE, 23.0 cd A^−1^, 12.3 lm W^−1^ and 6.7%. Moreover, the increased maximum luminance together with the decreased TPBi component is shown in Device A-E, and it is opposite to the turn-on voltage. It is known that the hole mobilities of mCP is 1.2 × 10^−4^ cm^2^ V^−1^ s^−1^, while the electron mobility of TPBi is 3.3 × 10^−5^ cm^2^ V^−1^ s^−1^ ^30–33^. Therefore, the unbalance of charge becomes worse when the content of TPBi is enhanced. And it is worth mentioning that the Device E achieved maximum luminance of 15800 cd m^−2^, which is one of best luminance in printed OLEDs^7,8,16^.

The Device B, F, G and H with the same structure were designed to study the influence of spin-coating and printing process on device performance. The maximum CE, PE and EQE of double layers spin-coated Device F are 24.2 cd A^−1^, 12.8 lm W^−1^ and 7%, which are little better than those of the best single layer printed Device B. The Device H with double printed layers shows the maximum luminance of 2192 cd m^−2^, CE of 10.9 cd A^−1^, PE of 7.6 lm W^−1^, and EQE of 3.2%. Generally speaking, the device performance gets worse with the printed layer increasing.

In addition, to prove the benefit of blended host to the device efficiency, the Device I has fabricated with spin-coating PEDOT:PSS and EML (mCP: Ir(mppy)_3_) with the ratio of 90:10. The Device I has the same structure with Device F except for host. Compared with 24.2 cd/A of Device F, Device I exhibits the poorer efficiency of 17.1 cd/A. The same situation happens in printed EML devices of Device B and E. So it proves that the blended host is beneficial to the device efficiency. It is noteworthy that the maximum efficiencies of Device I happen on 1500 cd/m^2^, so the CE, PE and EQE of Device I at 100 cd/m^2^ are poorer than those at 1000 cd/m^2^.

**Figure 4:**
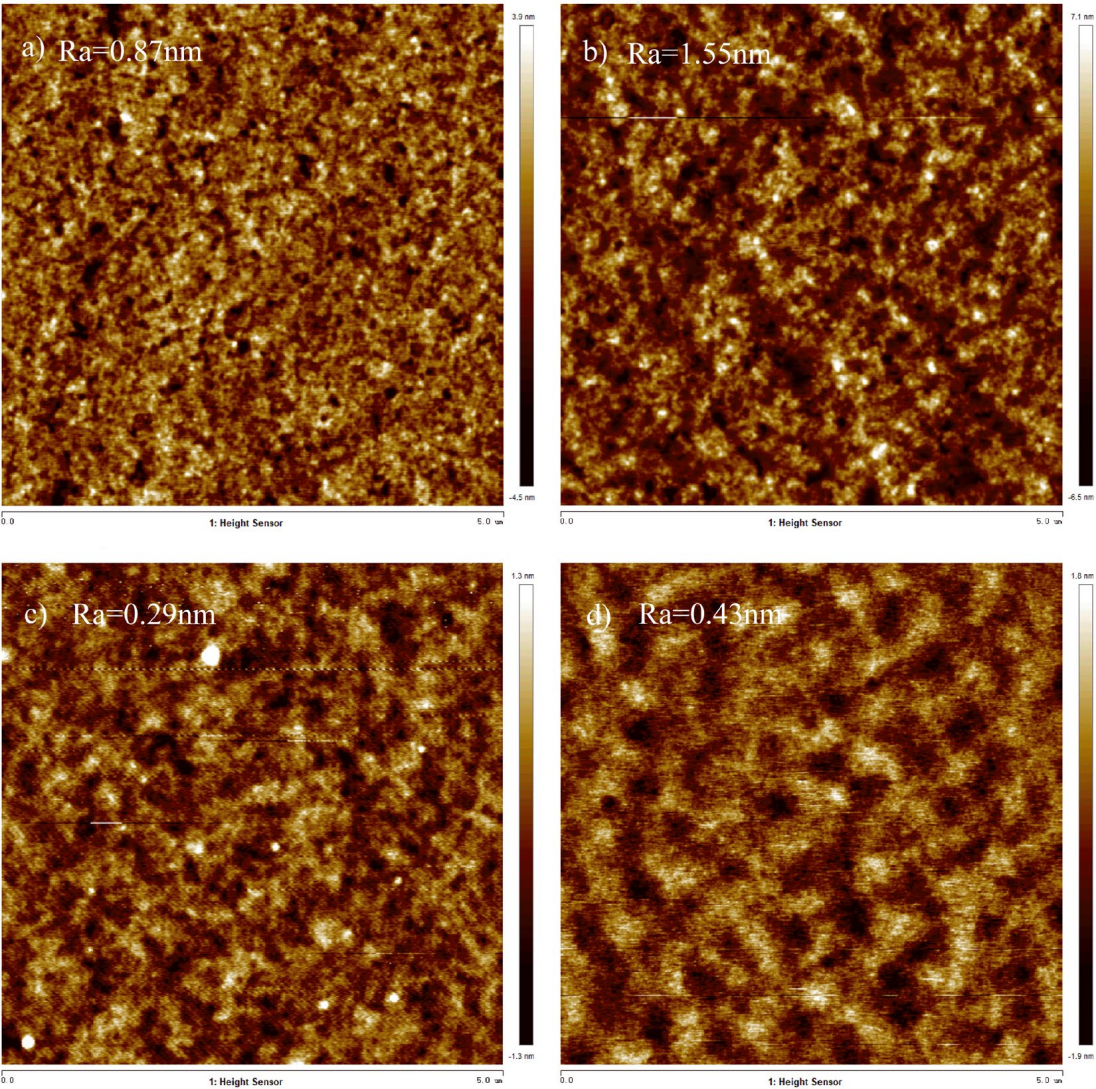
The AFM images of (**a**) spin-coated HTL; (**b**) printed HTL; (**c**) spin-coated EML; (**d**) printed EML.

To investigate the difference of device performance between the spin-coated and inkjet printed OLEDs, the film properties of PEDOT:PSS and EML have been researched. From the atomic force microscopy (AFM) images of different films as shown in Fig. 4, it is found that the surface roughness of spin-coated HTL, printed HTL, spin-coated EML, and printed EML (ink of EML-2) are 0.87, 1.55, 0.29, and 0.43 nm, respectively. In general, spin-coating can achieve better film morphology than that of inkjet printed film.

Moreover, Kelvin force microscopy (KFM) recorded different work functions, 4.45, 4.52, 4.62, and 5.06 eV in spin-coated HTL, printed HTL, spin-coated EML, and printed EML films, as shown in Table 4. The work function of spin-coated HIL is lower than that of printed, which means a lower surface potential barrier from ITO to HIL. Similarly, a high work function of printed EML will cause big potential barrier between HIL and EML. The work functions of single material film were shown in Table S2.

**Table 4:** The performance of different films.

	Ra (nm)	Work function (eV)	Contact angle
Spin-coated PEDOT:PSS	0.87	4.45	20.1°
Printed PEDOT:PSS	1.55	4.52	28.7°
Spin-coated EML	0.29	4.62	75.8°
Printed EML	0.43	5.06	86.1°

**Figure 5:**
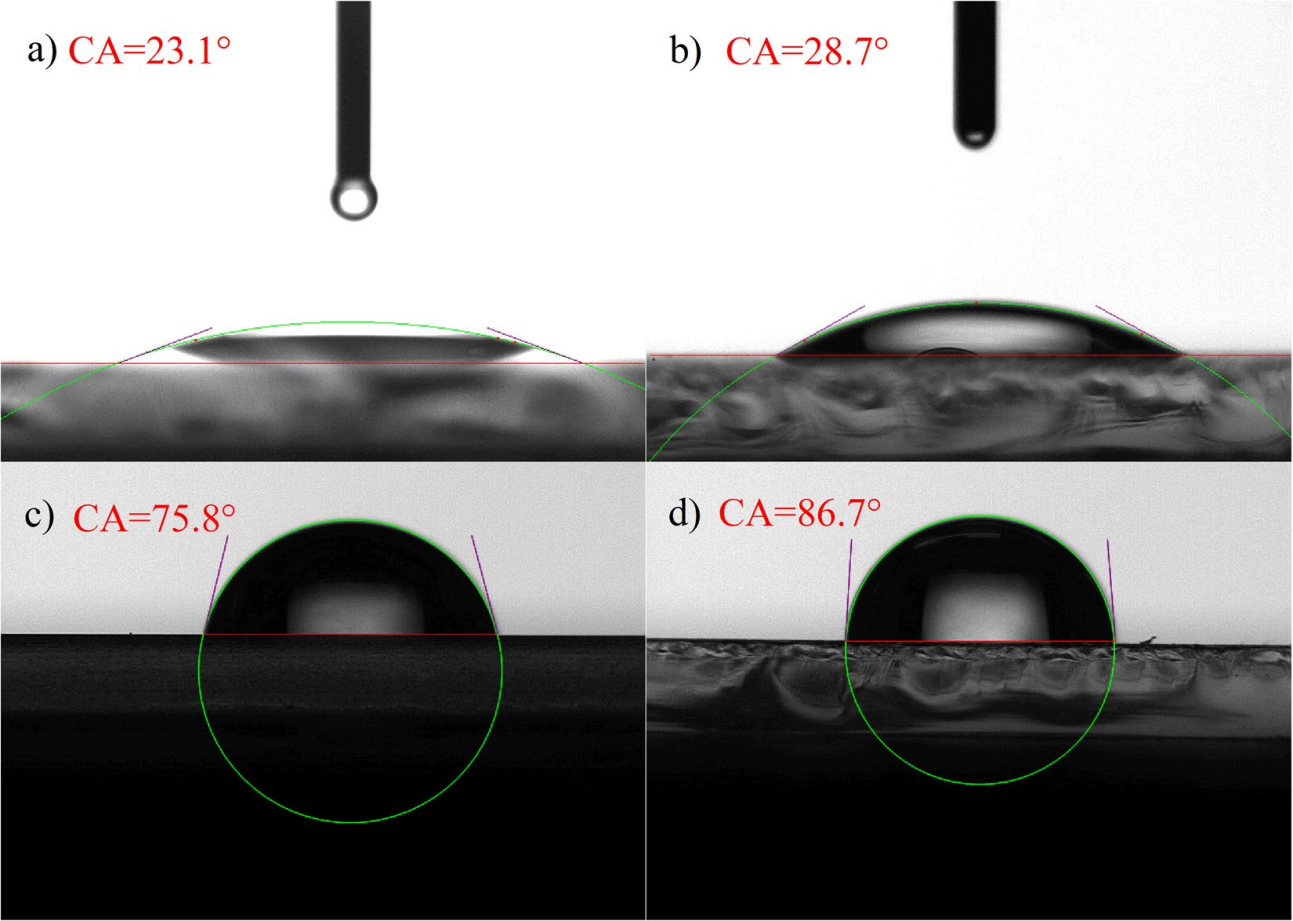
The contact angles of water on (**a**) spin-coated PEDOT:PSS; (**b**) printed PEDOT:PSS; (**c**) spin-coated EML; (**d**) printed EML.

The contact angles of spin-coated PEDOT:PSS, printed PEDOT:PSS, spin-coated EML, and printed EML were determined to be 23.1, 28.7, 75.8, and 86.7°, as shown in Fig. 5. Because the inks were experienced different forces in spin coating and inkjet printing processes, different arrangement of molecular may happen, which results in different contact angles^34,35^. The spin-coated PEDOT:PSS has smaller contact angle than the printed film, suggesting that the spin-coated PEDOT:PSS has better wettability for the solution deposition of next layer^36^. Hence, a better interface between functional layers is expected.

**Figure 6:**
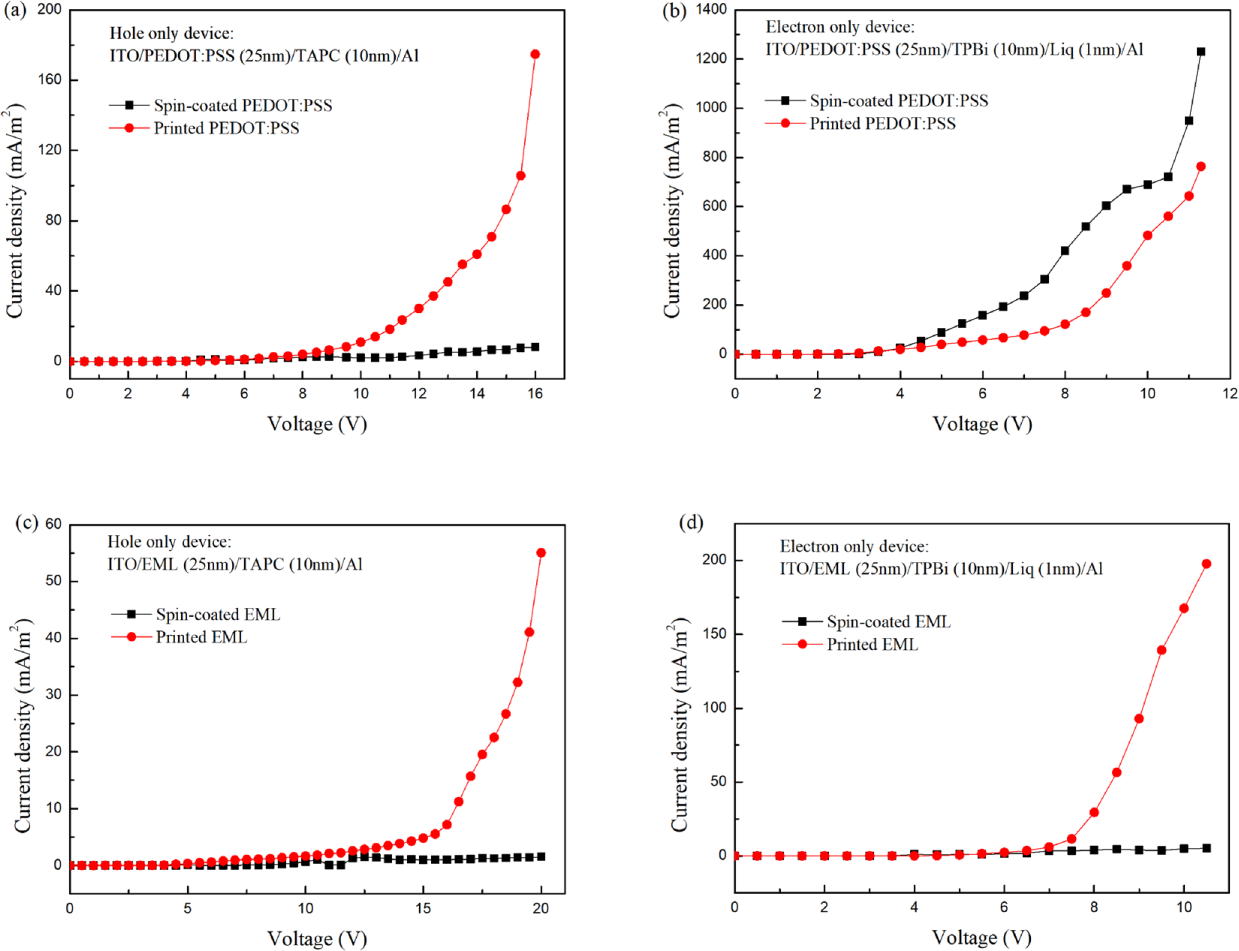
The V-J curves of single carrier devices.

The performance difference between the spin-coated and inkjet printed OLEDs is also due to difference of carrier transport ability of HTL and EML. The hole and electron only devices were made with the structure of ITO/test layer (25 nm)/TAPC (10 nm)/Al and ITO/test layer (25 nm)/TPBi (10 nm)/Liq (1 nm)/Al and the results are shown in Fig. 6a,b. It reveals that the hole mobility of printed PEDOT:PSS is higher than that of the spin-coated one, and the opposite is true for the electron mobility. As for the EML, the hole and electron transport abilities of printed are higher than the spin-coated in Fig. 6c,d. The high carrier mobility of double layer printed device leads to the lowest turn on voltage, as shown in device H. On the other hand, a low carrier mobility of spin-coated EML can confine the exciton to stay in EML, which contributes to the high efficiency realized in device F.

As shown in Table 5, the printed OLED in the last few years are listed. In contrast to the spin-coated OLEDs, the research on printed ones is rare. As shown in Table 5, the most of devices were characterized with single layer printed, low luminance and high turn on voltage (>4 V). Except for the devices mentioned in Table 5, some efficient printed OLEDs without complete detail data were not listed. In contrast, the devices in this work exhibit low turn-on voltage, high luminance and high efficiency, and even the double layer printed OLED shows CE of 10.9 cd/A and luminance of 2129 cd/m^2^.

**Table 5:** The summary of printed OLED.

	Printed layers	Layers^a^	V_on_	Current efficiency (cd/A)	EQE (%)	EL peaks (nm)	Luminance (cd/m^2^)
Device B	EML	4	4.0	23	6.7		2314
Device E	EML	4	3.7	15.9	4.6		15800
Device H	HTL + EML	4	3.5	10.9	3,2		2192
Ref.^7^	EML	6		10.73		468 (0.14, 0.25)	2600
Ref.^8^	EML	4	4.5	0.61			760
Ref.^14^	HTL	5	4.5	11.7			
Ref.^16^	HTL	5		24		516	~1000
Ref.^17^	HTL + EML	2		(0.43 lm/W)			137
Ref.^37^	EML	6	~4.5	45	13.9	550	~1000
Ref.^38^	EML	4	5.5	0.71		464	289

^a^The layers in OLED except for anode and cathode.

## Conclusions

The idea of conventional co-evaporation of multi-materials for OLEDs has been applied to solution processing to solve the interface issue happened in solution deposition of multilayer OLED devices. Maximum current efficiency (CE) of 24.2 cd A^−1^ and external quantum efficiency (EQE) of 7.0% have been achieved for spin-coated device with mCP:TPBi as the host. Maximum CE and EQE of 23.0 cd A^−1^ and 6.7% have been achieved for inkjet-printed device. The roughness of spin-coated films is a little better than the printed ones. Owing to larger contact angle, the spin-coated PEDOT:PSS shows better wettability for the solution deposition of next layer than the printed one. There is a ~0.4 eV gap of work function between spin-coated and printed EMLs, which may lead to the difference of injection barrier. At the same time, the spin-coated EML exhibits low electron and hole mobilities, which could confine exciton to get high efficiency. All of those factors contribute to the performance difference between spin-coated and inkjet-printed devices.

## Experimental Section

### General information

The viscosities of solvents were measured by Kinexus Lab of Malvern. And the surface tension was tested by Ez-Pi plus of Kibron Inc. UV–vis absorption spectra were recorded on a PerkinElmer LAMBDA 750 spectrophotometer. PL spectra were measured on a Hitachi F-4600 fluorescence spectrophotometer. Atomic force microscopy (AFM) and Kelvin force microscopy (KFM) measurements were recorded by using a Dimension ICON Scanning Probe Microscope at ambient temperature. Highly ordered pyrolytic graphite, whose work function in air is 4.6 eV, was taken as the reference. The EMLs for KFM measurement was deposited on the spin-coated PEDOT:PSS layer. The contact angles were tested by using a contact angle meter model SL150 (USA KINO Industry). The organic emitting films were printed by a Dimatix 2850 printer with the nozzle diameter of 21 μm. The volume of a single ink drop is approximately 10 pL.

### Materials

PEDOT:PSS (poly(3,4-ethylenedioxythiophene):poly-(styrenesulfonate)) (VPAi 4083, Heraeus), 4,4′,4″-tris[3-methylphenyl(phenyl)- aminotriphenylamine (m-MTDATA), 1,1-bis((di-4-tolylamino)phenyl)cyclohexane (TAPC) and 1,3-bis(carbazolyl)benzene (mCP) were purchased from Xi’an Polymer Light Technology Corp. 1,3,5-tri(phenyl-2-benzimidazoly)-benzene (TPBi), tris[2-(p-tolyl)pyridine]iridium(III) (Ir(mppy)_3_), and 8-hydroxyquinolatolithium (Liq) were purchased from Shanghai Han Feng Chemical Co., Ltd. All materials were without further purification.

### OLED fabrication and measurements

The OLED devices were fabricated with a structure of ITO/PEDOT:PSS (25 nm)/emissive layer (25 nm)/TPBi (30 nm)/Liq (1 nm)/Al (100 nm). The ITO substrates with 10 Ω ▯^−1^ were pre-cleaned *via* a routine procedure and treated by O_2_ plasma for 3 min. A layer of PEDOT:PSS was deposited on the ITO substrate *via* spin-coating or ink-jetting to form a hole transporting layer. The PEDOT:PSS-coated substrates were baked in an oven at 120 °C for 15 min. The solvent for spin-coating EML is CB. The structure of TPBi (30 nm)/Liq (1 nm)/Al (100 nm) was thermally deposited in sequence in a vacuum chamber at a base pressure of less than 6 × 10^−4^ Pa. The device performance (EL spectra, J-V curves, L-V, and EQE values) was measured with a Spectra Scan PR655 and a computer controlled Keithley 2400 Source. All measurements were carried out at room temperature under ambient conditions. EQEs of the devices were calculated from the luminance, current density and the EL spectrum, assuming a Lambertian distribution.

## Supplementary information

Supporting information
