# Supplementary material for: Blended host ink for solution processing high performance phosphorescent OLEDs
Source: Sci Rep. 2019 May 2;9:6845. doi: 10.1038/s41598-019-43359-4 (PMC6497667; doi:10.1038/s41598-019-43359-4)
Supplement: Supplementary file 1 — Supporting information [file 41598_2019_43359_MOESM1_ESM.docx]

Blended host ink for solution processing high performance phosphorescent OLEDs`

Tong Lin^1,2^, Xue Sun^1,2^, Yongxu Hu^3^, Wanying Mu^4^, Yuling Sun^1^, Dongyu Zhang^1*^, Zisheng Su^5^, Bei Chu^2^, Zheng Cui^1^

^1^ Printable electronics research center, Suzhou Institute of nanotech and nano-bionics, Chinese Academy of Sciences, Suzhou 215123, Jiangsu, PR China

^2^ State Key Laboratory of Luminescence and Applications, Changchun Institute of Optics, Fine Mechanics and Physics, Chinese Academy of Sciences, Changchun 130033, PR China

^3^ School of Chemical Engineering, University of Science and Technology Liaoning (USTL), Anshan, 114051, PR China

^4^ College of Materials Science and Engineering , Shanghai University, Shanghai 200444, PR China

^5^ College of Physics and Information Engineering, Quanzhou Normal University, Quanzhou 362000, PR China

Table S1 The design and performance of OLED devices

|  | PEDOT:PSS | EML | Maximum CE/PE/EQE (cd A^-1^/lm W^-1^/%)^a^ |
| --- | --- | --- | --- |
| Device B | Spin-coating | Printing mCP:TPBi:Ir(mppy)_3_ (45:45:10) | 23.0/12.3/6.7 |
| Device B1 | Spin-coating | Printing m-MTDATA:TPBi:Ir(mppy)_3_ (45:45:10) | 5.3/3.6/1.5 |
| Device B2 | Spin-coating | Printing TAPC:TPBi:Ir(mppy)_3_ (45:45:10) | 4.5/2.9/1.4 |





Figure S1 (a) V-J-L curves, (b) J-CE curves, (c) J- PE curves and (d) J-EQE of Device B, B1 and B2.





Figure S2 The AFM images of printing (a) m-MTDATA:TPBi:Ir(mppy)_3_ and (b) TAPC:TPBi:Ir(mppy)_3_ films.

Table S2 The work functions of single material film.

|  | Spin-coated mCP | Printed mCP | Spin-coated TPBi | Printed TPBi |
| --- | --- | --- | --- | --- |
| work function (eV) | 4.32 | 4.34 | 4.55 | 4.59 |
